# Genetic background of cognitive decline in Parkinson's disease

**DOI:** 10.3389/fcogn.2024.1379896

**Published:** 2024-07-22

**Authors:** Antonela Blazekovic, Kristina Gotovac Jercic, Sabina Devedija, Fran Borovecki

**Affiliations:** ^1^Department for Functional Genomics, Center for Translational and Clinical Research, University of Zagreb School of Medicine, University Hospital Center Zagreb, Zagreb, Croatia; ^2^Department for Anatomy and Clinical Anatomy, University of Zagreb School of Medicine, Zagreb, Croatia; ^3^Department of Neurology, University Hospital Center Zagreb, University of Zagreb School of Medicine, Zagreb, Croatia; ^4^Department of Neurology, University Hospital Dubrava, Zagreb, Croatia

**Keywords:** Parkinson's disease, genes, cognition, polygenic score, MAPT, SNCA, APOE, GBA

## Abstract

Parkinson's disease (PD) is a complex disorder that is influenced by multiple genetic risk factors. There is a significant heterogeneity in PD presentation, both pathologically and clinically. Some of the most common and important symptoms affecting the patient are cognitive impairment and dementia. However, the genetic and biological basis underlying the differences in cognitive profiles, including the development of dementia in PD, is not yet well understood. Understanding the role of genes in cognitive outcomes is crucial for effective patient counseling and treatment. Research on familial PD has discovered more than 20 genes that can cause the disease. The identified genes responsible for familial cases of PD are *LRRK2, PARK7, PINK1, PRKN*, or *SNCA* gene, although there may be other genes that also contribute. Additionally, some of these genes may also play a role in cases that were previously thought to be sporadic. Currently, numerous well-described genes increase the risk of cognitive decline in PD, each with varying levels of penetrance. The aim of this review is to identify the relevant genetic factors that contribute to differences in cognition. We discuss the genes that may affect cognition and the challenges in establishing a clear genetic diagnostic and prognostic assessment. This article aims to demonstrate the complexity of the genetic background of cognition in PD and to present the different types of genotype changes that can impact cognition through various neurobiological mechanisms.

## 1 Introduction

Parkinson's disease (PD) is a neurodegenerative disorder primarily affecting motor function, however, cognitive impairment is also a common and highly disabling feature of PD. The presentation and progression of cognitive symptoms in PD are highly variable. Some patients may begin to develop cognitive changes in the prodromal phase of the disease. In contrast, others may evade any noticeable deficits in cognitive functioning for more than 20 years after symptom onset. Various risk factors have been identified for the development of cognitive dysfunction in PD. These factors include age, disease duration, and severity of motor symptoms. Although age is considered a primary risk factor, the genetic and biological mechanisms that contribute to the heterogeneity in cognitive profiles and the onset of dementia in PD, remain largely unknown.

Patients with PD may experience cognitive impairment and dementia as a result of other underlying conditions, such as Alzheimer's disease (AD) or cerebrovascular disease. These conditions can present with symptoms that overlap with those of PD dementia, making it challenging to identify the exact cause of dementia clinically. Definitive biomarkers are not yet available for differentiating the types of dementia. In clinical research, it is crucial to differentiate between the types of dementia because treatments target the abnormal accumulation of alpha-synuclein, the pathological hallmark of PD dementia and dementia with Lewy bodies (DLB) (Lindström et al., 2014), or tau (Anand and Sabbagh, 2015) or amyloid-β (Delrieu et al., 2014), which underlies AD. There are many similarities between different diseases in terms of cognitive symptoms and neuropathologically. The most common neuropathological feature of PD dementia is the advanced limbic and neocortical Lewy pathology (Lindström et al., 2014). However, amyloid-β and tau pathologies related to AD are also frequent and independently associated with cognitive impairment in PD (Smith et al., 2019) reflecting the complex genetic background. In line with the duality of neuropathology, established genetic risk loci for cognitive progression in PD include genes implicated in PD risk and Lewy pathology, as well as AD susceptibility loci (Tunold et al., 2023b).

In this review, we discuss the genes that might impact cognition and point out the methodological difficulties in establishing a precise genetic diagnosis and result forecast. Our primary objective is to illustrate the intricate genetic basis of cognition in PD and to present the diverse array of genotype variations that may affect cognition.

## 2 Genetic background: What we know?

Cognitive performance and decline in individuals with PD are heterogeneous, and genetics may explain some of this variability. Numerous studies have been carried out to investigate the relationship between PD and cognitive impairment, with most focusing on the development of dementia. Based on the available evidence, it appears that some genes, namely apolipoprotein E (*APOE*) (Mata et al., 2014), microtubule-associated protein tau (*MAPT*) (Williams-Gray et al., 2009), glucocerebrosidase (*GBA*) (Liu et al., 2021) and alpha-synuclein (*SNCA*) (Nagy et al., 2007; Kéri et al., 2008; Kochunov et al., 2017), may contribute to the susceptibility of cognitive impairment in PD. This is not surprising, given the overlap of neuropathology and symptomatology with other conditions such as AD and dementia with Lewy bodies. Studies have shown different genetic structures in patients with Lewy body dementia (DLB) depending on the extent of accompanying AD-pathology (Van Der Lee et al., 2021; Kaivola et al., 2022). It has been shown that *APOE* was specifically associated with the AD-positive group, and *GBA* with the group of DLB patients without AD elements (Tunold et al., 2023a). Investigations in other genes, including leucine-rich repeat serine/threonine-protein kinase 2 (*LRRK2*) (Hong et al., 2017), catechol-O-methyltransferase (*COMT*) (Paul et al., 2016), brain-derived neurotrophic factor (*BNDF*) (Białecka et al., 2014), transmembrane protein 108 (*TMEM108*) (Liu et al., 2021), WW domain-containing oxidoreductase (*WWOX*) (Liu et al., 2021), the mitochondrial E3 ubiquitin protein ligase 1 (*MUL1*) (Jo et al., 2021) have led to conflicting results.

**Figure 1:**
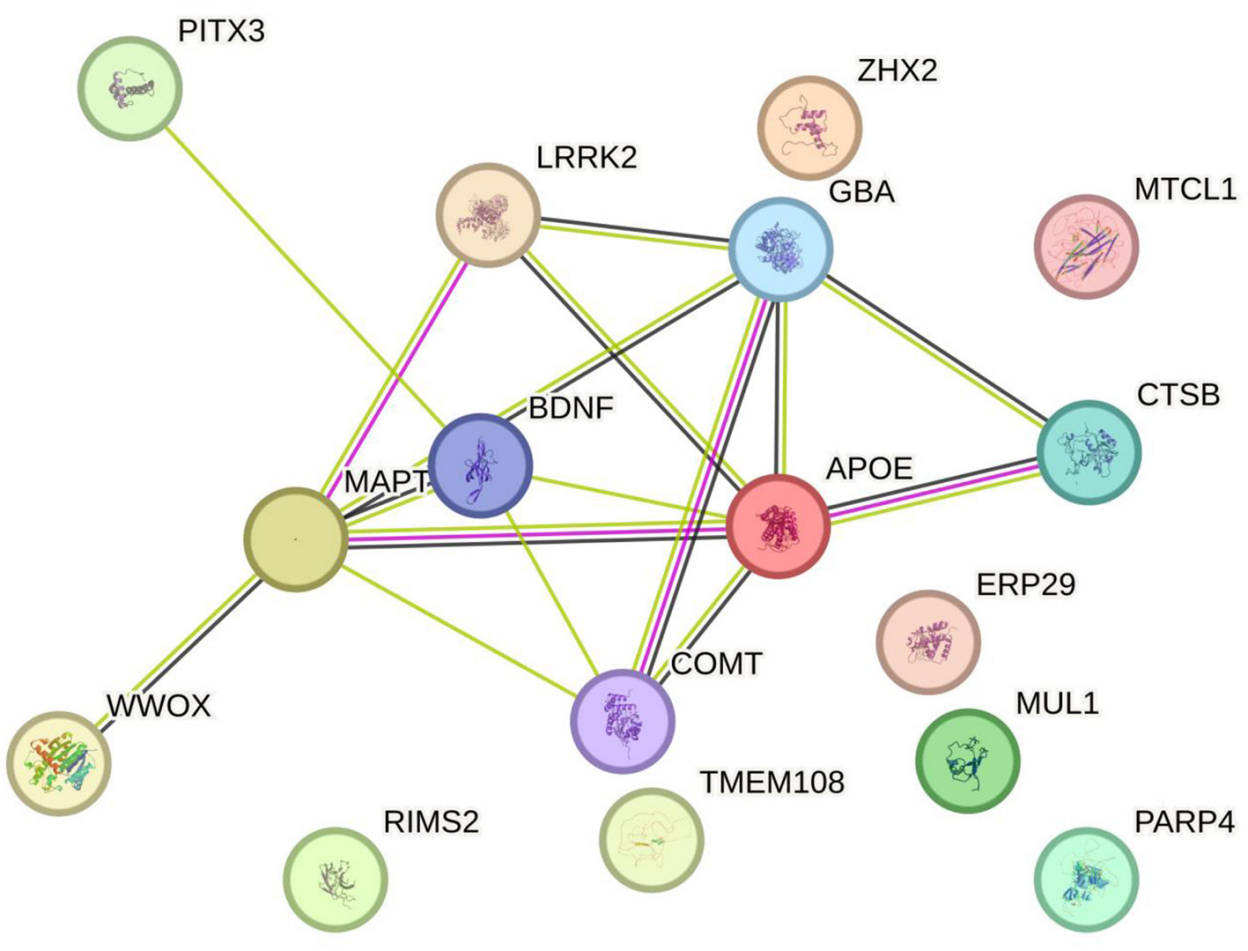
A visual representation of all the genes that have been identified in the literature as potentially linked to cognitive symptoms in PD and discussed in this review (string.org).

This review covers a selection of genes that have been associated with cognitive symptoms in PD, ranging from those with the most hits in literature searches to those mentioned in only one study. Figure 1 displays all the genes reviewed in this article.

## 3 Methods

**Figure 2:**
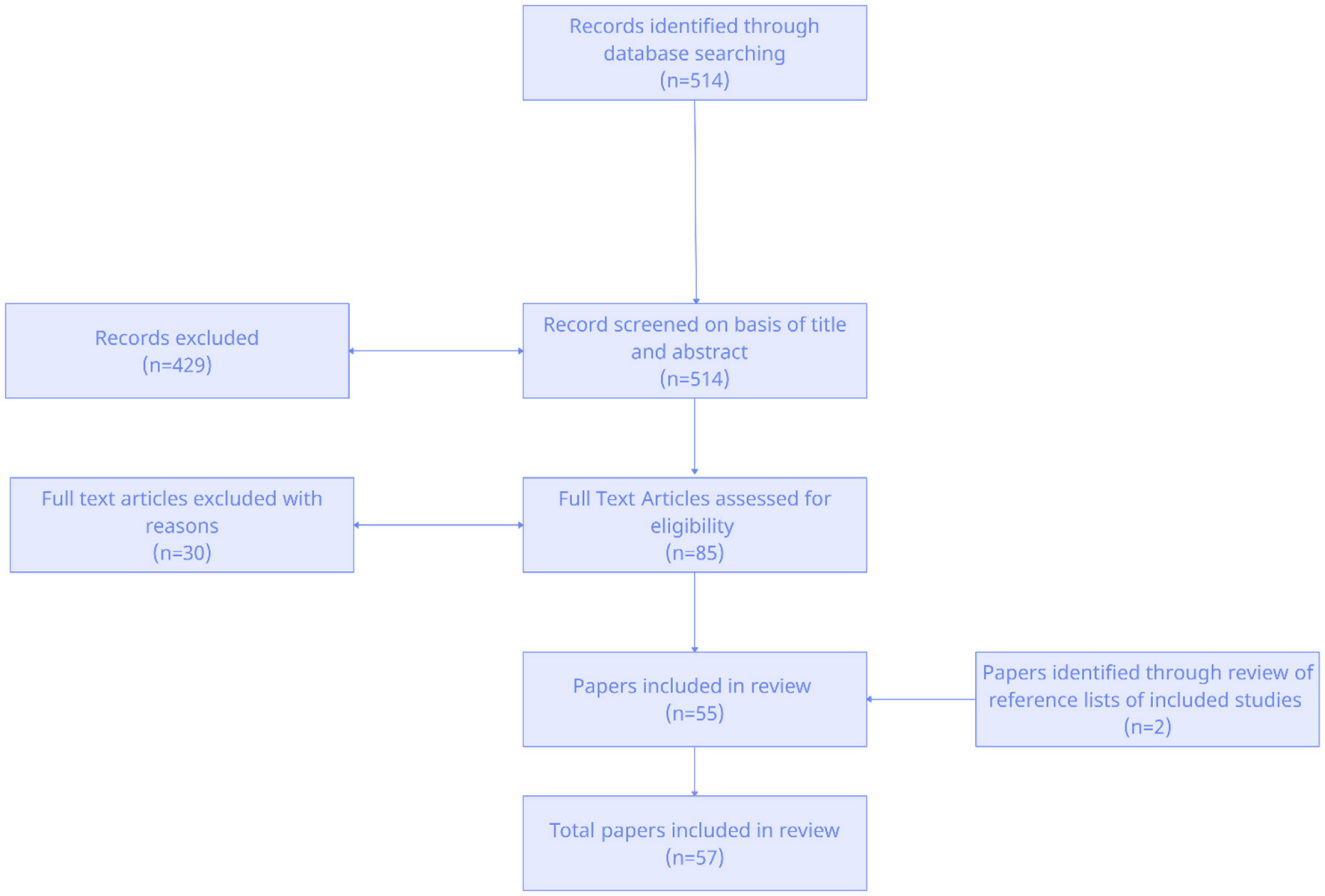
Literature review flowchart.

The literature review aimed to identify genes that have been associated with cognitive symptoms in PD, ranging from those with the most hits in literature searches to those mentioned in only one study. Figure 2 summarizes the reviewing process. Search terms used are listed in Supplementary Table 1. Criteria for inclusion were: specific inclusion of people with dementia or cognitive impairment; suggesting or explaining gene involvement; and identifying a specific research study or reviewing a collection of studies.

## 4 Results

A literature search was conducted to find studies that investigated the link between genetic variation and cognitive outcomes in patients with PD. The search strategy is outlined in Figure 2. The search yielded a total of 514 records, out of which 429 were eliminated as irrelevant based on the title and abstract. The remaining 85 articles were read in full. This led to a final selection of 55 relevant articles. In addition, a manual search through the reference lists of these papers uncovered two more publications of interest, bringing the total number of included articles to 57.

Most literature hits were found for APOE, SNCA, MAPT, and GBA. However, the studies have varied greatly in their design, sample size, candidate genes, and cognitive outcome measures. This has led to partly conflicting conclusions drawn from the reported results. Some studies have retrospectively assessed dementia diagnosis from medical records, while others have used advanced neuropsychological testing.

### 4.1 APOE

Polymorphisms in the *APOE*gene are associated with AD. These polymorphisms are also linked to cognitive decline in both healthy individuals and patients with PD (Tsuang et al., 2013; Mata et al., 2014; Paul et al., 2016). The ε4 variant of *APOE* (*APOE* ε4) gene is the best-known risk factor for AD (O'Donoghue et al., 2018), however, there has been uncertainty regarding how APOE E2 and E4 variants may impact cognitive impairment and other overlapping features of neurodegeneration. In a study performed on 1,079 PD patients, Mata, and colleagues found that the presence of *APOE* ε4 allele was linked to decreased cognitive performance across various domains such as memory, attention/executive function, and language processing (Mata et al., 2014). However, in patients without dementia, the ε4 allele only affected total learning and semantic verbal fluency (Mata et al., 2014). Interestingly, studies show that the *APOE* ε4 allele increases the likelihood of developing dementia in the context of synucleinopathy, even without any other contributing factors (Tsuang et al., 2013). Additionally, the fact that the ε4 allele occurs more frequently in cases with a low overall brain neuritic plaque burden suggests that *APOE* might contribute to neurodegeneration through mechanisms that are not associated with amyloid processing (Tsuang et al., 2013). Moreover, it is shown that the relationship between *APOE* ε4 and cognitive changes is significantly modified by age in most cognitive domains (Liu et al., 2023). Individuals in the elderly group carrying the *APOE* ε4 allele experience a steeper decline in global cognition and most cognitive domains (*p* = 0.001) (Liu et al., 2023). They also had a higher probability of experiencing cognitive deterioration compared to non-carriers (Liu et al., 2023). However, no significant correlations between *APOE* ε4 and cognitive decline were observed in the middle-aged group. These findings indicate that the *APOE* ε4 allele has an age-dependent impact on cognitive decline in PD patients, despite the lack of clear underlying mechanisms.

There is a whole spectrum of cognitive changes that APO e4 is connected to. For instance, APOE ε4 is associated with poorer cognition, particularly the early symptoms of memory, language, and attention (He et al., 2024). Furthermore, APOE ε4 is associated with elevated NO levels, which is linked to impaired attention and executive function (He et al., 2024). A study by Sakurai et al. (2024) showed that APOE E4 carriers with slow gait had the lowest global cognitive performance and smaller gray matter volumes compared to non-APOE E4 carriers with normal gait. The coexistence of APOE E4 and slow gait best predicted global and domain-specific poorer cognitive performances, mediated by smaller gray matter volume (Sakurai et al., 2024). Also, data from the Honolulu Heart Program/Honolulu-Asia Aging Study showed that people who retained relatively high levels of cognitive functioning until death were more likely to have the presence of the APOE ε2 allele, while people who demonstrated significant cognitive waning several years before death had more likely the APOE ε4 allele (Margrett et al., 2022). A genome wide association study (GWAS) which included clinical data for 3364 patients with 12,144 observations, showed that the APOE ε4 tagging variant, rs429358, was significantly associated with composite and cognitive progress in PD (Tan et al., 2021). Another GWAS from 12 longitudinal cohorts in a total of 4,093 patients with 22,307 observations showed the same APOE E4 tagging variant being associated with greater cognitive deficits in patients (Iwaki et al., 2019). A lot more studies also support the growing body of research that the APOE ε4 allele can be used as a predictor of cognitive decline in PD patients (Morley et al., 2012; Monsell et al., 2014; Paul et al., 2016).

However, it should be emphasized that the impact of *APOE* is not unequivocally related to PD. Some studies do not show an association between *APOE* variants and cognitive decline in PD. In a German ongoing observational multicenter cohort study (LANDSCAPE study) including 447 PD patients *APOE* ε4 allele was not associated with a diagnosis of cognitive impairment in PD (PD with mild cognitive impairment and PD with dementia) or with deficits in specific neuropsychological domains (Mengel et al., 2016). Also, Gan et al. showed that the presence of APOE ε4 allele is associated with hyperhidrosis and depression, but not global cognition, activities of daily life, motor function and other neuropsychitric symptoms in patients with dementia with Lewy bodies (Gan et al., 2022). No, association is shown also in a cohort of 237 patients diagnosed with PD with and without dementia (Pierzchlińska et al., 2018). The APOE genotypes were not significantly associated with longitudinal changes in individual cognitive domains, however, carriers of the APOE-ε4 allele were shown to be at increased risk of mild cognitive impairment and dementia within the study period (OR 3.03; *p* = 0.006) (Chung et al., 2021).

Various study results highlight that the influence of APOE variation on cognition is complex, in some cases varying based on diagnosis and possibly underlying disease pathology. A large study performed on 514 individuals with various neurodegenerative disorders assessed the influence of APOE carrier status and disease cohort on performance across five cognitive domains (Dilliott et al., 2021). Regardless of the disease group, it has been shown that individuals with the E4 allele have lower verbal memory and visuospatial performance than those with the E3/3 allele, while individuals with the E2 allele did not show any significant difference in cognitive performance (Dilliott et al., 2021). However, individuals with the E2 allele and frontotemporal dementia have significantly poorer performance than those with the E3/3 allele in the visuospatial, attention/working memory, and executive function domains (Dilliott et al., 2021). Also, there is the question of potential sex-specific effects of APOE ε4 on cognitive decline. In the sex-stratified GLME models adjusted for covariates, Kim et al. showed that males with APOEε4 had a steeper rate of cognitive decline than those without APOEε4, in contrast, there was no such significant interaction in females (Kim et al., 2021). There is also the possibility of an interaction effect on brain structure between the APOE and other genotypes. Sampedro et al. showed that cortical atrophy was associated with harboring the APOE ε4 allele only in BDNF val/met subjects (both in control and PD groups) (Sampedro et al., 2019).

### 4.2 SNCA

The *SNCA* gene encodes alpha-synuclein, a soluble protein expressed at presynaptic terminals in the central nervous system and plays a crucial role in the pathogenesis of PD. In the context of cognitive decline in PD, *SNCA* has been implicated in contributing to the development of cognitive impairment and dementia (Nagy et al., 2007; Kéri et al., 2008; Kochunov et al., 2017). Increasing evidence suggests that *SNCA* plays an important role in regulating dopamine release within the mesolimbic dopamine system, a critical pathway for the modulation of many behavioral and emotional processes. Due to depleted dopamine levels, PD patients with *SNCA* risk haplotypes tend to have less efficient stimulus-reward learning (Kéri et al., 2008). This is similar to the learning patterns observed in patients with unmedicated PD (Nagy et al., 2007). However, the inefficient learning in patients with *SNCA* risk haplotypes is much more pronounced than in healthy participants with the same risk haplotypes. Furthermore, the *SNCA*-rs356181 variant is shown to contribute to axonal injury and myelin damage in the anterior corona radiata. This area contains associative fibers connecting to the frontal lobe, which are associated with processing speed and working memory (Kochunov et al., 2017). On the other hand, a study on around a thousand PD patients revealed no association of variant *SNCA* rs356219 with cognitive decline (Tsuang et al., 2013). A longitudinal study of SNCA gene promoter (SNCA-Rep1) investigated Rep1 polymorphism and longitudinal change in cognition in early PD (Tan et al., 2022). They determined Rep1 allele lengths (“long” and “short”) in 204 early PD patients and showed significant decline in executive function in long Rep1 allele carriers vs. short allele carriers (Tan et al., 2022). Another study showed a similar effect, long Rep1 allele carriers had significantly lower Mini-Mental State Examination (MMSE; *p* = 0.010) and higher UPDRS Part III (*p* = 0.026) and H & Y (*p* = 0.008) scores compared to short allele carriers (controlled for age, sex, and disease duration) (Ng et al., 2019). In the analysis performed by Jo and al, SNCA rs11931074 was determined to be most significantly associated with PD (odds ratio = 0.66, 95% confidence interval = 0.56–0.78, *p* = 7.75 × 10^−7^), however there was no correlation with dementia. In the PD group, only MUL1 nucleotide polymorphism (SNP) rs3738128 (odds ratio = 2.52, 95% confidence interval = 1.68–3.79, *p* = 8.75 × 10^−6^) was found to be most significantly associated with dementia in PD (Jo et al., 2021). In a study aimed to predict global cognition in PD with machine learning using structural neuroimaging, genetics and clinical and demographic characteristics they found the rs894280 of SNCA gene as the most novel finding of ML (Ramezani et al., 2021). *Post-hoc* analysis revealed a robust association between rs894280 and GC, attention, and visuospatial abilities (Ramezani et al., 2021). Cognitive impairment was reported in all monogenic PD forms with variable rates (58.8% PINK1, 53.9% SNCA, 50% DJ1, 29.2% VPS35, 15.7% LRRK2 and 7.4% Parkin) (Piredda et al., 2020). Although monogenic forms make up only a 10th of all PD patients, it is important to show that cognitive decline is dependent on the type of mutation.

### 4.3 MAPT

Tau is a microtubule-associated protein involved in microtubule assembly and stabilization. Variants in *MAPT* gene have been firmly established as a risk factor for progressive supranuclear palsy and corticobasal degeneration (Galpern and Lang, 2006). Abnormalities of tau protein play a central role in the development of progressive supranuclear palsy. Meanwhile, haplotype variation in the tau gene MAPT can impact the risk of PD and PD dementia. There have been several studies examining the relationship between *MAPT* haplotypes and cognitive performance in PD, yielding conflicting results. According to published data, there is a hypothesis that suggests tau and alpha-synuclein to be involved in shared or converging pathways in the development of PD (Goris et al., 2007). Data suggests that tau inversion may influence the cognitive impairment and dementia that some patients with idiopathic PD experience (Goris et al., 2007). They found a synergistic interaction between the *MAPT* inversion polymorphism SNP rs356219 from the 3′ region of *SNCA*. Longitudinal follow-up of a subset of 109 incident PD cases suggested that *MAPT* is a genetic risk factor that plays a role in the early development of cognitive impairment and dementia in PD (Goris et al., 2007). Furthermore, Williams-Gray et al. conducted a study showing that individuals with PD and the H1/H1 haplotype experience a higher cognitive decline and are more susceptible to dementia compared to those with the H2 haplotype (Williams-Gray et al., 2009). In contrast, Paul et al. observed no discernible association between the *MAPT* H1 haplotype and changes in cognitive functioning over time within a PD cohort (Paul et al., 2016). Notably, both studies accessed cognitive function only via MMSE scores. Studies employing a more comprehensive array of cognitive measures have failed to identify any significant impact of the *MAPT* H1 haplotype on cognitive function within PD cohorts (Mata et al., 2014). For instance, an investigation involving a substantial PD cohort did not report a significant correlation between the *MAPT* H1 haplotype and outcomes across nine distinct psychometric tests evaluating cognition (Nagy et al., 2007; Kéri et al., 2008; Kochunov et al., 2017). Similarly, a study on an essential tremor cohort revealed no influence of *MAPT* haplotypes on cognitive performance (Ghanem et al., 2023). On the other hand, a study was conducted to evaluate the relationship between common genetic variations in the SNCA (rs11931074, rs894278) and MAPT (rs242557_H1c haplotype, rs3744456) genes and the severity and duration of both motor and cognitive performance (Alcalay et al., 2010). The results showed that increased severity of cognitive function was associated with MAPT (H1c haplotype, *p* = 0.05) (Alcalay et al., 2010). A study investigating the neurocognitive correlates of *MAPT* haplotypes using functional magnetic resonance imaging showed that H1 homozygosity was associated with impaired picture recognition memory in PD patients and control subjects (Winder-Rhodes et al., 2015). Their results revealed that H1 homozygotes with PD showed additional age-related differences in blood-oxygen-level-dependent response in the medial temporal lobes (Winder-Rhodes et al., 2015). The ICICLE-PD study, established to define the characteristics and prevalence of cognitive change soon after PD diagnosis, showed that neurocognitive deficits are common even in recently diagnosed patients and that the associated regional brain activations are influenced by genotype (Nombela et al., 2014).

It should be noted that the relationship between cognitive function and certain diseases can vary over time. For example, in a study examining the connection between baseline levels of amyloid β1–42 (Aβ42), total tau (t-tau), phosphorylated tau (p-tau) in cerebrospinal fluid (CSF), and cognitive performance, no correlation was found (Liu et al., 2015). However, after starting treatment with levodopa, higher levels of p-tau and p-tau/Aβ42 were found to predict a decline in cognitive performance involving memory and executive functions (Liu et al., 2015). Rittman and colleagues analyzed functional brain networks and found a correlation between the expression of MAPT in certain regions of the brain and the loss of connectivity in those regions in patients with PD (Rittman et al., 2016). The study also showed that the impairment of executive cognition was proportional to the loss of hub connectivity (Rittman et al., 2016). These findings demonstrate a connection between the regional expression of MAPT and the selective vulnerability of functional brain networks to neurodegeneration (Rittman et al., 2016).

### 4.4 GBA

Mutations in the *GBA* gene have been demonstrated to be a strong risk factor for PD, but have also been associated with greater cognitive decline in PD (Liu et al., 2021). In a study conducted on 699 PD patients with age at onset below 51 years, it was observed that individuals carrying *GBA* mutations (N370S or L444P), reported cognitive impairment more frequently than those with no such mutations (Alcalay et al., 2010). However, MMSE data did not confirm this difference (Alcalay et al., 2010). Another study found that *GBA* mutation carriers performed worse than noncarriers on the Montreal Cognitive Assessment (MoCA) (Brockmann et al., 2011). In an observational longitudinal study cohorts from North America and Europe representing 2,304 patients with PD were followed for up to 12.8 years (median, 4.1) with a total of 20,868 in-person study visits (Liu et al., 2016). This study showed that PD patients with *GBA* variants presented with accelerated cognitive decline over time compared to other PD patients (Liu et al., 2016). A study assessing cognitive outcomes after deep brain stimulation of the subthalamic nucleus (STN-DBS) showed that *GBA* mutations are associated with early cognitive decline following STN-DBS (Mangone et al., 2020). A mouse model of Gaucher disease that exhibited memory deficits and progressive accumulation of alpha-synuclein/ubiquitin aggregates in hippocampal neurons was also studied (Sardi et al., 2011).

### 4.5 Other genes of potential interest

There has been a lot of discussion about certain genes being linked to cognitive function in PD. However, there is no conclusive evidence to support this claim as many studies have failed to establish a connection. This is particularly true when analyzing specific genes or variants, as a large cohort of patients is required. For instance, a study conducted on 500 patients with PD by the NeuroGenetics Research Consortium found no correlation between psychotic symptoms and any of the examined polymorphisms in the apolipoprotein, alpha-synuclein, or microtubule-associated protein tau genes (Factor et al., 2011). However, a GWAS study confirmed the association of mutations in *GBA* and *APOE* with dementia in PD (Tan et al., 2021), but interestingly reported also the regulating synaptic membrane exocytosis 2 (*RIMS2*) genes as a progression locus [*p* = 2.78 × 10^−11^; hazard ratio (HR) = 4.77], as well as suggestive associations in transmembrane protein 108 (*TMEM108*; HR = 2.86, *p* = 2.09 × 10^−8^) and WW domain-containing oxidoreductase (*WWOX*; HR = 2.12, *p* = 2.37 × 10^−8^) genes in PD, but with limited effect sizes (Liu et al., 2021). In a microarray analysis on 634 PD patients, the mitochondrial E3 ubiquitin protein ligase 1 (*MUL1*) SNP rs3738128 (odds ratio = 2.52, 95% confidence interval = 1.68–3.79, *p* = 8.75 × 10^−6^) was found to be most significantly associated with dementia in PD suggesting an essential role of mitochondrial dysfunction in the development of dementia in patients with PD (Jo et al., 2021). SNPs in zinc fingers and homeoboxes 2 (*ZHX2*) and endoplasmic reticulum resident protein 29 (*ERP29*) genes were also associated with dementia in PD (Jo et al., 2021). Soutar et al. used a mitophagy screening assay to assess the functional significance of risk genes identified through GWAS (Soutar et al., 2022). They found that two regulators of PINK1-dependent mitophagy initiation, KAT8 and KANSL1, modulate lysine acetylation and contribute to PD (Soutar et al., 2022). KANSL1 is located on chromosome 17q21 and has long been considered to be associated with the MAPT gene. While the study does not exclude the possibility of an association between MAPT and PD, it provides strong evidence that KANSL1 plays a crucial role in the disease (Soutar et al., 2022).

SNPs in the Paired Like Homeodomain 3 (*PITX3*) gene have also been identified as risk factors for PD (relative risk of 1.4 and estimated population attributable risk of 0.21 for the C allele of rs2281983) (Fuchs et al., 2009). A Swedish prospective study on 133 PD patients showed that PD patients carrying the *PITX3* C allele had an increased risk of developing dementia (hazard ratio: 2.87, 95% CI: 1.42–5.81, *p* = 0.003), compared to the PD patients homozygous for the T-allele. Furthermore, the *PITX3* C allele carriers with PD had poorer cognitive performance in the visuospatial domain (*p* < 0.001) and a higher incidence of visual hallucinations. A trend toward a lower striatal DAT uptake in the *PITX3* C allele carriers was suggested, but could not be confirmed (Bäckström et al., 2017). A study on 299 PD patients, consisting of 23 carriers and 276 non-carriers of *LRRK2* G2385R, concluded that the *LRRK2* G2385R genotype may not be associated with cognitive dysfunction in PD (Hong et al., 2017). Similarly, other *LRRK2* variants showed no correlation, i.e. cognitive functions were similarly affected in PD patients with and without *LRRK2* G2019S mutation with mainly impaired visuospatial and executive abilities (Ben Sassi et al., 2012). One study even showed that G2019S mutation status is associated with better attention, executive function, and language fluency in PD patients (Alcalay et al., 2015).

A case-control study was conducted in the Chinese population to investigate the genetic link between AD, PD, and cognitive impairment (Wang et al., 2022). The study evaluated the association of 13 single-nucleotide polymorphisms in 9 genes (BIN1, CLU, ABCA7, CR1, PICALM, MS4A6A, CD33, MS4A4E, and CD2AP) known as AD GWAS top hits, with both PD and cognitive function in PD patients (Wang et al., 2022). A total of 454 controls and 442 PD patients were included in the study. The study did not find any significant association between the susceptibility loci for AD and PD cases, PD-dementia or PD-mild cognitive impairment (Wang et al., 2022). Based on the findings, it can be inferred that the 13 single-nucleotide polymorphisms from AD genome-wide association studies may not have a significant role in the genetic predisposition of PD and cognitive function in the Chinese population. Similarly, a study aimed to examine the association between genome-wide significant loci of Type 2 Diabetes Mellitus (T2DM) and the risk of PD and AD as well as the severity of cognitive impairment (Chung et al., 2015). They included 500 PD patients, 400 AD patients, and 500 unrelated controls and analyzed 32 genetic variants from 11 genes associated with T2DM: CDC123, CDKAL1, CDKN2B, FTO, GLIS3, HHEX, IGF2BP2, KCNJ11, KCNQ1, SLC30A8, and TCF7L2 (Chung et al., 2015). The CDC123 SNP rs11257655 was found to be associated with MMSE score < 26, and the CDKN2B SNPs (rs2383208, rs10965250, and rs10811661) were found to be associated with MoCA score < 26 in PD patients, however, these associations were not statistically significant after Bonferroni correction (Chung et al., 2015). Other genetic variants had no association with the risk of PD or AD and the severity of cognitive impairment. Finally, a large-scale exploratory genetic analysis of cognitive impairment in PD in which 1,105 PD patients from the PD Cognitive Genetics Consortium were genotyped for 249,336 variants showed that 13 genomic regions were associated with at least one decline in one cognitive domain (Mata et al., 2017). These included *GBA* rs2230288, *PARP4* rs9318600 and rs9581094 and *MTCL1* rs34877994 (Mata et al., 2017).

### 4.6 Polygenic risk score

Polygenic scores (PGS) are a way of combining the risk associated with multiple genetic variants into a single score. This is done by calculating the combined effects of individual genotypes, using data from large-scale GWAS. In other words, PGS adds up the weighted risks of every genetic trait to generate an overall score. A large meta-analysis comprising together 17,02 PD patients found four PGS significantly associated with cognitive decline: intelligence (*p* = 5.26e−13), cognitive performance (*p* = 1.46e−12), educational attainment (*p* = 8.52e−10), and reasoning (*p* = 3.58e−5) (Faouzi et al., 2024). On the other hand, a multicenter longitudinal cohort of 143 PD patients showed no correlation between PD PGD and impulse control disorders (Ihle et al., 2020). Tunold et al. (2023b) have emphasized that lysosomal variants can have a significant impact on cognitive decline in patients with PD who are less likely to develop AD. Additionally, their findings suggest that stratification by the polygenic burden of AD risk alleles can help us better understand the genetic factors that contribute to cognitive impairment in PD patients (Tunold et al., 2023b). A study on 225 patients with Gaucher disease (including 199 without PD and 26 with PD) has shown that the PD PGS is higher in individuals who carry the *GBA* gene compared to those who do not (Blauwendraat et al., 2023). Variants located near *CTSB* and *SNCA* may also have a potential gene-gene interaction with *GBA* (Blauwendraat et al., 2023). This suggests that common risk variants may affect the underlying biological pathways, as the variants included in the PD genetic risk score were more frequent in patients with Gaucher disease who developed PD. A recent study examined the genetic architecture of AD, schizophrenia, and PD through the use of PGSs, and found that hallucinations in patients with PD were associated with the genetic architecture of AD, particularly with the *APOE* gene (Kusters et al., 2020). The study also noted some potential associations between hallucinations and the genetic architecture of schizophrenia, as well as with genetic susceptibility for PD in late-onset patients (Kusters et al., 2020).

Finally, a recent study examined the relationship between PGS and disease progression in a longitudinal population-based cohort of patients with PD (Paul et al., 2018). The study found that the PGS, based on established PD GWAS risk loci, is associated with cognitive decline and supports previous findings linking these risk factors to motor symptom progression (Paul et al., 2018). The results suggest that these genetic risk factors may contribute to disease progression in multiple domains. The study supports the idea that PD is influenced by multiple genetic variants, each with a small effect size.

## 5 Discussion

The symptoms and progression of cognitive decline in PD can vary widely. Cognitive impairment and dementia are common disorders in PD. Around 80% of PD patients are expected to develop dementia within 10 years of diagnosis. PD is a complex disorder that is influenced by multiple genetic risk factors. Therefore, it is crucial to understand the role of genes in cognitive outcomes to provide accurate treatment and counseling to patients. Currently, multiple genes are strongly associated with PD and increase its risk, although they have varying degrees of penetrance. Additionally, several loci in our DNA have been identified through large, genome-wide association studies that also increase the risk of PD. In this review, we provide a summary of the current knowledge about each of the seven genes that are strongly linked to PD, along with their respective relationships to cognition. However, our understanding of the underlying mechanisms and effective treatments for these disorders remains limited. Given the complexity of the symptoms in PD, it is unlikely that there is a single genetic cause. Although some studies have provided promising results, research focusing on individual genes can only explain a small portion of the variation in cognitive impairment in PD. Most studies investigating cognitive impairment in PD have employed candidate gene approaches, examining genes such as *GBA, LRRK2, MAPT*, and *SNCA*. However, results have often been inconclusive or not replicated independently.

This literature demonstrates a clear association only between *GBA* mutations and increased risk of cognitive decline and dementia. However, for other genes, there are conflicting results. The connection between *APOE* variation and cognition is complex, and it may vary based on diagnosis and underlying disease pathology, as well as sex and age. There can also be an interaction effect on brain structure between the *APOE* and other genotypes. There is a possible relationship between *SNCA* variability and the cognitive profile in PD, although the details of this relationship remain incompletely explored. This association is complex, rs356181 is shown to be connected with axonal injury while there is no association of some other *SNCA* variants with cognitive decline. Machine learning using structural neuroimaging, genetics and clinical and demographic characteristics found the rs894280 of *SNCA* gene as the most novel finding. There have been multiple studies that have examined the connection between *MAPT* haplotypes and cognitive performance in PD. However, the results obtained have been conflicting. While some studies have indicated a relationship between the H1 haplotype and faster cognitive decline, others have not found any such connection. Nonetheless, studies that conducted multimodal analysis or attempted to identify more subtle changes than just the MMSE have revealed that the relationship is much more complex. It has also been observed that the relationship between cognitive function and specific diseases can vary over time and can be influenced by other factors.

There has been a lot of debate regarding the potential correlation between certain genes and cognitive function in PD. However, there is no conclusive evidence to support this claim. Many studies have failed to establish a connection, as a large number of patients is required to draw meaningful conclusions. GWAS studies have significantly improved our understanding of the genetic risk factors for PD by identifying several genetic variants. They offer a partial solution to this problem by examining markers across the entire genome simultaneously. Furthermore, studies have shown that patients' cognitive decline is linked to the accumulation of genetic risk factors for PD, as determined by calculating a polygenic risk score.

However, to better understand the disease biologically, it is essential to comprehend the functional importance of these risk loci. In conclusion, cognitive impairment is an important aspect of PD that can greatly impact a patient's quality of life. By identifying the risk factors and molecular mechanisms that contribute to this symptom, we can develop better prognostics and treatments to improve outcomes for patients with PD.

## Author contributions

AB: Conceptualization, Visualization, Writing – original draft. KG: Writing – review & editing. SD: Writing – review & editing. FB: Writing – review & editing.
