# Supplementary material for: Genetic background of cognitive decline in Parkinson's disease
Source: Front Cognit. 2024 Jul 22;3:1379896. doi: 10.3389/fcogn.2024.1379896 (PMC13281075; doi:10.3389/fcogn.2024.1379896)
Supplement: Supplementary file 1 [file Data_Sheet_1.PDF]

Supplementary tables

Table 1 Search terms used in the review

(("parkinson s disease"[All Fields] AND "cognition"[All Fields]) AND (("genes"[All Fields] OR "genetics"[All Fields]) AND (humans[Filter]))
("parkinson s disease"[All Fields] AND "cognition"[All Fields]) AND (("genes"[All Fields] OR "genetics"[All Fields]) AND (humans[Filter])) AND "APOE"[All Fields]
("parkinson s disease"[All Fields] AND "cognition"[All Fields]) AND (("genes"[All Fields] OR "genetics"[All Fields]) AND (humans[Filter])) AND "SNCA"[All Fields]
("parkinson s disease"[All Fields] AND "cognition"[All Fields]) AND (("genes"[All Fields] OR "genetics"[All Fields]) AND (humans[Filter])) AND "MAPT"[All Fields]
("parkinson s disease"[All Fields] AND "cognition"[All Fields]) AND (("genes"[All Fields] OR "genetics"[All Fields]) AND (humans[Filter])) AND "GBA"[All Fields]
("parkinson s disease"[All Fields] AND "cognition"[All Fields]) AND (("genes"[All Fields] OR "genetics"[All Fields]) AND (humans[Filter])) AND "GWAS"[All Fields]
("parkinson s disease"[All Fields] AND "cognition"[All Fields]) AND (("genes"[All Fields] OR "genetics"[All Fields]) AND (humans[Filter])) AND " Polygenic scores "[All Fields]
